# A gene edited pig model for studying LGR5^+^ stem cells: implications for future applications in tissue regeneration and biomedical research

**DOI:** 10.3389/fgeed.2024.1401163

**Published:** 2024-06-06

**Authors:** Amanda B. T. Hill, Yanet M. Murphy, Kathryn M. Polkoff, Laura Edwards, Derek M. Walker, Adele Moatti, Alon Greenbaum, Jorge A. Piedrahita

**Affiliations:** ^1^ College of Veterinary Medicine, North Carolina State University, Raleigh, NC, United States; ^2^ Comparative Medicine Institute, North Carolina State University, Raleigh, NC, United States; ^3^ Joint Department of Biomedical Engineering, University of North Carolina at Chapel Hill, and North Carolina State University, Raleigh, NC, United States

**Keywords:** LGR5, gene edited pigs, organ stem cells, regenerative medicine, animal models

## Abstract

Recent advancements in genome editing techniques, notably CRISPR-Cas9 and TALENs, have marked a transformative era in biomedical research, significantly enhancing our understanding of disease mechanisms and helping develop novel therapies. These technologies have been instrumental in creating precise animal models for use in stem cell research and regenerative medicine. For instance, we have developed a transgenic pig model to enable the investigation of LGR5-expressing cells. The model was designed to induce the expression of H2B-GFP under the regulatory control of the LGR5 promoter via CRISPR/Cas9-mediated gene knock-in. Notably, advancements in stem cell research have identified distinct subpopulations of LGR5-expressing cells within adult human, mouse, and pig tissues. LGR5, a leucine-rich repeat-containing G protein-coupled receptor, enhances WNT signaling and these LGR5^+^ subpopulations demonstrate varied roles and anatomical distributions, underscoring the necessity for suitable translational models. This transgenic pig model facilitates the tracking of LGR5-expressing cells and has provided valuable insights into the roles of these cells across different tissues and species. For instance, in pulmonary tissue, Lgr5^+^ cells in mice are predominantly located in alveolar compartments, driving alveolar differentiation of epithelial progenitors via Wnt pathway activation. In contrast, in pigs and humans, these cells are situated in a unique sub-basal position adjacent to the airway epithelium. In fetal stages a pattern of LGR5 expression during lung bud tip formation is evident in humans and pigs but is lacking in mice. Species differences with respect to LGR5 expression have also been observed in the skin, intestines, and cochlea further reinforcing the need for careful selection of appropriate translational animal models. This paper discusses the potential utility of the LGR5^+^ pig model in exploring the role of LGR5^+^ cells in tissue development and regeneration with the goal of translating these findings into human and animal clinical applications.

## Introduction

Regenerative medicine has made significant advancements towards enhancing the body’s natural repair processes, thereby providing alternative treatments for diseases that were previously incurable (Golchin et al., 2020; Altyar et al., 2023). Although pharmacological therapies benefit many patients, there remains a range of pathologies that do not respond well to drug-based therapies, thus urgently requiring new therapeutic solutions. Examples include myocardial infarction, peripheral vascular disease, partial tissue loss (burns, large fractures, etc.), and various forms of organ failure, such as renal failure (Altyar et al., 2023). In response, there has been a focus on the study of cells, tissues, and their products for their utilization in the repair and restoration of diseased cell functionality (Golchin et al., 2020). This research has led to the exploration of diverse technologies, notably stem cell therapy and gene editing.

A paramount challenge in this field is the generation of promising preclinical data that can lead to the successful clinical application of these technologies. To overcome this hurdle, there has been a shift from small to large animal models to thoroughly test the safety and efficacy of the developed products (Ribitsch et al., 2020). This transition is crucial in translational medicine, where selecting an appropriate animal model is key to bridging the gap between scientific discoveries and clinical applications.

The LGR5 receptor, present in stem cells across various tissues, relies on niche-specific signaling for cell fate determination (Leung et al., 2018). Extensive research has explored the receptor’s roles in different tissues, and it is widely acknowledged that the Wnt/R-spondin pathway is a major signaling mechanism influencing the behavior of cells expressing this receptor. LGR5^+^ cells are integral to tissue homeostasis (Jaks et al., 2008; Muñoz et al., 2012), regeneration (Carpino et al., 2012; Rodriguez et al., 2021), and even cancer (Xu et al., 2019). In mice, using gene edited, Lgr5^+^ cells have been identified in various tissues, including the intestinal epithelium, pancreatic ductal cells, lungs, prostate gland, hair follicles, eyes, cochlea, tongue, and others (Leung et al., 2018). It has been shown in multiple tissues including gastrointestinal, lung, cochlea, and skin that LGR5^+^ cells have the ability to generate organoids with multiple cell types (Sato et al., 2011; Koo and Clevers, 2014; Mclean et al., 2017; Polkoff et al., 2022a; Schaaf, et al., 2023). Additionally, as will be described below in more detail, others have shown that LGR5 cells have regenerative capacity on a wide range of tissue and can participate in tissue repair after injury and during homeostasis.

Although many studies have been performed in mice with the goal of elucidating the role behind the LGR5 receptor in different niches, an understanding of the role of LGR5^+^ cells in tissue development, homeostasis, regeneration, and disease in other species is lacking. The distinct anatomy and physiology of mice has led to results that, while highly valuable for understanding basic mechanisms, have drawbacks for translation, especially in terms of making progress towards discovering the therapeutic potential of LGR5^+^ cells. Unfortunately, the lack of reliable antibodies for LGR5 presents a significant obstacle to investigation of LGR5 cell populations in other species, including pigs. While scRNAseq has allowed the study of these cells in a few organs of humans and mice (Zepp et al., 2021; Kadur et al., 2022) it does not allow isolation of the cells for further studies or for transplantation. Moreover, the costs associated with scRNAseq are such that it limits the number of samples that can be analyzed thus preventing an in-depth look of LGR5 cell properties, and this limits translation. At present, only in pigs and mice can LGR5 cells be isolated and as described previously the mouse, while an excellent model for basic sciences, has limitations for clinical translation.

This led to the generation of the LGR5-H2B-GFP line (Polkoff et al., 2022b), which has Histone 2B linked to GFP (H2B-GFP) under the control of the endogenous LGR5 promoter, allowing us to identify LGR5-expressing cells *in vivo* and *in vitro*. Due to regulatory requirements for the approval of new medical therapies, completely replacing animal models is not yet feasible. However, in line with the “3R” principle of replace, reduce, and refine (Ribitsch et al., 2020), the development of LGR5 transgenic pigs, which exhibit greater anatomical and physiological similarities to humans, represents a significant advancement. These models not only serve as refined alternatives but also contribute to the eventual reduction in the number of animals used in research. In fact, the reduction potential of this model is enormous. Experiments can be properly designed to investigate the impact of a treatment in multiple organ systems that contain LGR5^+^ cells, from the cochlea to the gastrointestinal tract, as the population of cells in question is both present and scientifically relevant in a vast number of tissues. Moreover, for developmental studies, in particular early development, pig fetuses can generate sufficient material for completion of experiments that would require a 5–10 times greater number of mouse fetuses. Furthermore, the mouse model requires hundreds, if not thousands of animals to generate data that may lack translational potential; the availability offered by the pig model described here allows to address those questions using a comparatively smaller number of animals.

Considering these factors, the goal of this manuscript is to present an overview of our findings using the LGR5-H2B-GFP model, highlighting the locations and distributions of LGR5^+^ cells across various tissues. Table 1 provides a comprehensive expression pattern of postnatal LGR5 in different tissues of mice, pigs, and humans. Understanding the specific tissue locations and function of LGR5^+^ cells is pivotal in assessing the translational potential of this model. Utilizing the LGR5-H2B-GFP model, we have been able to examine LGR5^+^ cells in diverse tissues, including the skin, cochlea, gastrointestinal tract, musculoskeletal system, and lungs, among others. In this review, we aim to summarize our key observations to date and shed light on the unique insights afforded by this model. We will delve into the current knowledge surrounding the LGR5 receptor in different organ systems and explore how the LGR5 transgenic model has expanded our understanding of the distribution and characteristics of these distinctive adult stem cells.

**TABLE 1:** Expression pattern of postnatal LGR5 in different tissues of mice, pigs, and humans.

Tissue/Organ	Expression pattern (mice)	Expression pattern (pigs)	Expression pattern (humans)
Gastrointestinal	Identified in the crypt base of intestines Barker et al. (2007), Pyloric region Barker and Clevers (2010), Injured pancreas Huch et al. (2015)	Identified in the crypt base of intestines Schaaf et al. (2023), base of the gastric antrum glands, biliary duct and gallbladder Pancreas	Identified in the Intestines Tsai et al. (2016), base of the gastric antrum glands Wu et al. (2013), biliary duct and gallbladder Zhang et al. (2023b)
Cochlea	Identified close to hair cells Bramhall et al. (2014) and after injury also close to hair cells Wang et al. (2015)	Identified in cochlear apex and base, high expression in Hensen cells Moatti et al. (2022)	Not available
Skin	Identified in the bulge region of hair follicles Polkoff et al. (2022b)	Identified in the bulge region of hair follicles Polkoff et al. (2022b)	Identified in the bulge region of hair follicles Polkoff et al. (2022b)
Lung	Identified in alveolar compartments Lee et al. (2017)	Identified adjacent to airway epithelium Polkoff et al. (2022a)	Identified adjacent to airway epithelium Polkoff et al. (2022a)
Musculoskeletal	Identified in tissues surrounding joints and tendons Feng et al. (2019)	Identified in tissues surrounding joints and tendons, anterior and distal patella, hoof, and head of fibula	Present in the deep zone of articular cartilage, associated with osteoarthritis Lee et al. (2021)
Eye	Identified in retina Chen et al. (2015)	Identified in retina Chen et al. (2015)	Identified in retina Curcio et al. (2015)

### LGR5^+^ cells in the gastrointestinal system

Organs in the gastrointestinal system, esophagus, stomach, small and large intestine, liver, pancreas, and gallbladder all express LGR5 and all derive from the embryonic foregut endoderm (Lemaigre, 2009). In mice, at the embryonic (E) E8.5 stage, gut endoderm starts expressing Lgr5, and this expression continues until E16.5, contributing to the development of the adult intestinal epithelium (Lugli et al., 2016; Tsai et al., 2016). Moreover, in both humans and mice, LGR5 plays a crucial role in endoderm induction. However, there are notable differences between these two species in terms of LGR5 expression. In humans, LGR5 expression is high in the endoderm and intestinal lineages, whereas LGR4 and LGR6 show lower levels of expression. Conversely, in mice, LGR4 and LGR6 are expressed at higher levels compared to LGR5 in these same lineages (Tsai et al., 2016).

LGR5 stem cells, initially identified in the small intestines and colon, have their roles most extensively studied within the gastrointestinal tract. In the intestinal lining, where LGR5 cells were first identified (Barker et al., 2007), they are located at the bottom of crypts, closely associated with Paneth cells. This unique stem cell niche is maintained primarily through Wnt secretion, with Paneth cells and myofibroblasts playing a key role in this process (Sato et al., 2011; Kabiri et al., 2014). In mouse intestines, as in humans and pigs, Lgr5 is present at the crypt base, where it can generate all cell types found on the villus (Sato et al., 2011; Schaaf, et al., 2023). Though Lgr5 does not appear crucial for the maintenance of healthy mouse intestinal epithelium, it is essential in tissue recovery post-injury. Studies have shown that after radiation-induced ablation of Lgr5^+^ cells, crypt regeneration is impeded (Metcalfe et al., 2014).

Regarding intestinal function, clear differences have been demarcated between humans, mice, and pigs (Tsai et al., 2016; Ishikawa et al., 2022; Schaaf, et al., 2023). Notably, in mice, Lgr4 expression surpasses that of Lgr5, whereas in human organoids, the situation is reversed, with LGR5 being dominant (Tsai et al., 2016). Another distinction is the cycling rate of LGR5^+^ cells; in humans, they are predominantly slow-cycling, contrasting with their faster-cycling murine counterparts (Ishikawa et al., 2022). This difference is further emphasized by the absence of OLFM4 in the mouse colon and the presence of LYZ, LGR5 and SOX9 expressing cells in the transit-amplifying/progenitor cell region in humans and pigs, but not in mice (Schaaf et al., 2023). In the crypt base, in contrast, cells co-express SOX9, LGR5 and LYZ in all three species (Schaaf et al., 2023).

**FIGURE 1:**
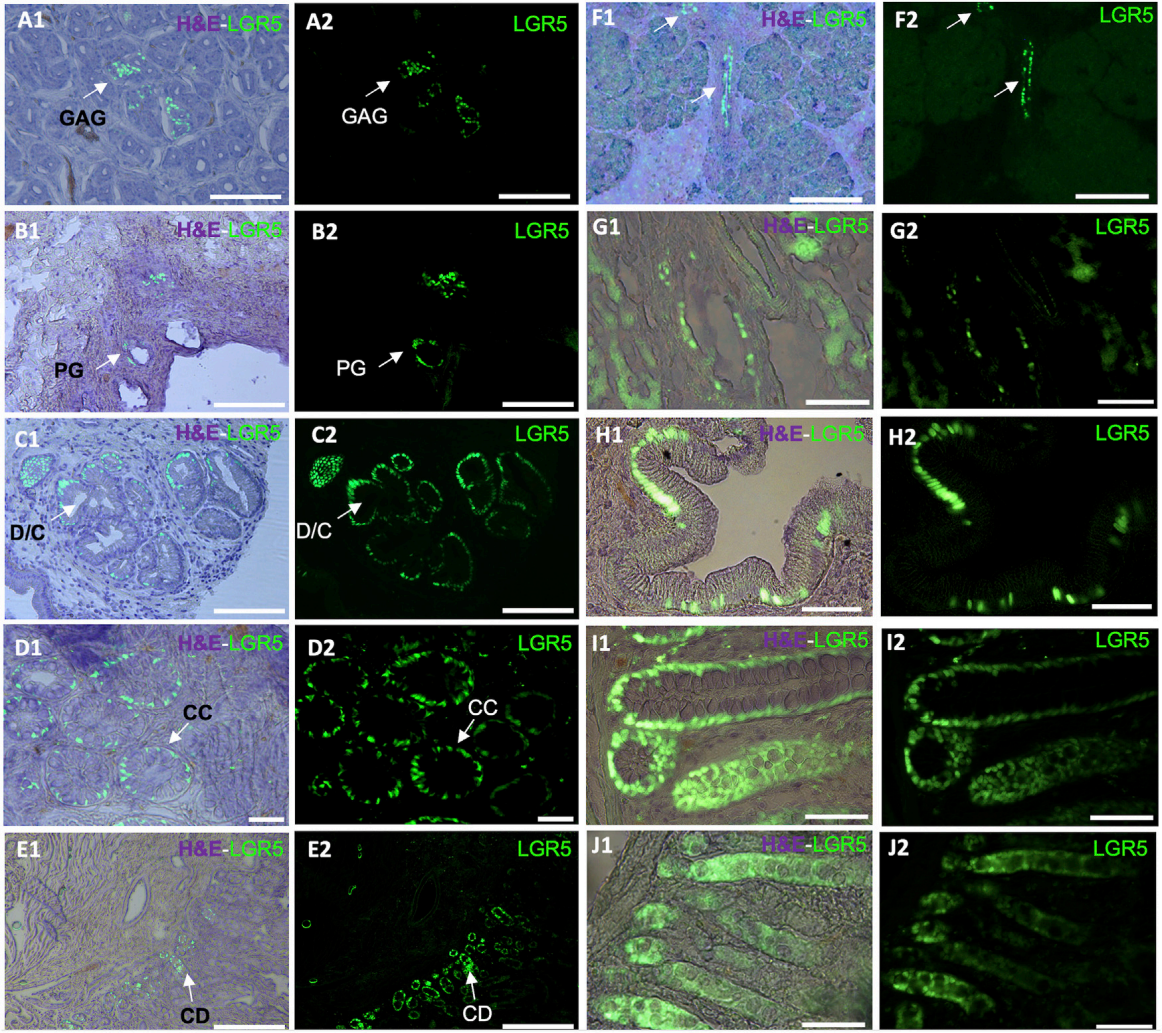
Detection of LGR5^+^ cells on organs of the pig gastrointestinal system. Anatomical location of the LGR5^+^ cells can be observed by expression of H2B- GFP and a hematoxylin and eosin (H&E) counterstain (A1–J1) in (A1–A2) LGR5^+^ cells the bottom of gastric antrum glands (GAG), Scale bar, 50 um. (B1–B2) LGR5^+^ cells in the bile duct peribiliary glands (PG). Scale bar, 50 um. (C1–C2) LGR5^+^ cells in the diverticula/crypts (D/C) of the gallbladder, Scale bars 25 um. (D1–D2) LGR5^+^ cells in the crypts of the colon. Scale bars, 25 um (CC). (E1–E2) LGR5^+^ cells in the crypt of the duodenum. Scale bar, 100 um (CD). (F1–F2) LGR5^+^ cells in the pancreas. Scale bar, 248 um. (G1–G2) LGR5^+^ cells in the liver. (H1–H2) LGR5^+^ cells in the gallbladder in higher magnification. (I1–I2) LGR5^+^ cells in the crypts of the colon in higher magnification. (J1–J2) LGR5^+^ cells in the crypts of the jejunum. Scale bars, 62.2 um.

Using the LGR5-H2B-GFP pig we demonstrated that, as in mice and humans, LGR5^+^ cells are located at the base of the crypt epithelium and can form enteroids *in vitro* (Schaaf et al., 2023). As depicted in Figure 1, LGR5^+^ cells are observable in the duodenum, colon and jejunum. A larger number of LGR5 expressing cells can be observed in the colon than in the other regions (Schaff et al., 2023). Moreover, isolated LGR5^+^ cells could generate organoids at high efficiency. The ability to culture the LGR5 cells allowed us to introduce mutations in the APC gene to develop a model of colon cancer, and injection of the mutated cells into the serosa of nude mice led to the generation of tumors, supporting the use of this model to study colon cancer progression and treatment (Schaaf et al., 2023).

The interactions between gastrointestinal Lgr5^+^ cells and the microbiome have also been a subject of significant research. For instance, in the stomach, *Helicobacter pylori* can induce chronic inflammation and this inflammation leads to the upregulation of stromal Rspo3, which influences the proliferation of Lgr5^+^ cells in the lower stomach region, but the impact of these hyperproliferation is still not clear (Sigal et al., 2017). Similarly, studies support a link between *Fusobacterium* nucleatum and colorectal tumors attributed to the increased presence of Lgr5^+^ cancer cells (Leung et al., 2018). This is confirmed in humans with LGR5/RSPO3 identified as tumor promoters with high levels associated with poor prognosis (Mehdawi et al., 2023). Due to its prognostic cancer value LGR5 cell has been referred to as the cancer stem cell (Montazer et al., 2023). Another burgeoning area of study within Lgr5^+^ research is cell metabolism. This is particularly relevant since different cell types exhibit distinct metabolic processes, influencing local tissue turnover rates. Intestinal stem cells, for example, have been observed to have a higher pyruvate-to-lactate ratio and respiration rate compared to Paneth cells. Recently the role of nutrients and metabolic pathways has shown that they can have a significant impact on intestinal LGR5 cells, but the mechanism is not yet understood (Shi and Wang, 2024).

In the murine stomach, Lgr5^+^ cells are less abundant and primarily located in the pyloric region in the gastric glands, pushing their progeny upward and influencing their differentiation into various epithelial cell types (Barker and Clevers, 2010). Although Lgr5^+^ cells originate from different sections of the neonatal mouse stomach epithelium, by 4 weeks post-birth, Lgr5 expression gradually fades from the stomach corpus (Barker and Clevers, 2010). As in the colon LGR5 has been associated with cancer, in this case, gastric adenocarcinomas (Wu et al., 2013). In our LGR5-H2B-GFP adult pig model, we have observed LGR5^+^ cells at the base of the gastric antrum glands (Figure 1A). This observation mirrors previous findings where LGR5^+^ cells were detected at the base of the human stomach (Wu et al., 2013).

In the adult liver, the Wnt pathway is active in hepatocytes and neighboring central veins (Planas-Paz et al., 2016) and Rspo3 is secreted by pericentral endothelial cells (Rocha et al., 2015). Yet, LGR5^+^ cells are normally not found in the homeostatic conditions. However, expression of Lgr5 can be upregulated after injury and Lgr5 expressing cells may play a role in liver regeneration via *de novo* generation of hepatocytes and ductal cells (Huch et al., 2015). Moreover, Lgr5^+^ cells could be isolated in both adult mice (Huch et al., 2015) and humans (Huch et al., 2015), and were able to form organoids retaining their bipotential characteristics, originating hepatocytes and cholangiocytes. Furthermore, recent studies focused on stimulating liver stem cell proliferation have increasingly focused on small soluble therapeutic molecules. It was suggested that Lgr5^+^ liver cells have the potential to be explored as an injury treatment for this organ and HGF/Rspo1 could be used to promote liver stem cell proliferation (Lin et al., 2017). In this context, to understand and explore the role of small molecules in stem cell niche fate, the animal model choice is extremely relevant, given that minor differences cause significant interspecies differences.

We recently reported that pigs and humans have a network of co-hepato/pancreatic stem/progenitor cells in the duodenum, the peribiliary glands of the intrahepatic and extrahepatic biliary trees, and in the pancreatic duct glands of intrapancreatic biliary trees, which supports hepatic and pancreatic regeneration postnatally (Zhang et al., 2023a). In our adult pig model, it is possible to observe LGR5^+^ cells in the biliary duct (Figure 1B) and gallbladder (Figures 1C,H). Similarly, as reported by Zhang et al. (2023b), biliary tree stem cells expressing LGR5 have been previously identified in humans and pigs and were found to co-express a variety of stem cells markers, including CD44, NIS, as well as early endodermal transcription factors, such as SOX9, SOX17, and PDX-1. Additionally, they were able to differentiate into hepatic (hepatocytes, cholangiocytes) and pancreatic (acinar cells, islets) cell populations. Similarly, large-scale production of human liver bipotential LGR5 stem cells has been reported opening the possibility of cell-based regenerative liver therapies (Schneeberger et al., 2020) emphasizing the need for a relevant animal model to develop the optimal clinical delivery methods.

Regarding the development of pancreatic tissue, in the mouse, at E14.5 and increasing by E17.5, Lgr5^+^ cells were observed in the bile duct lining and the ductal network. While in the adult mice, Lgr5 is normally not detected in the pancreas (Rodriguez et al., 2021), Lgr5 cells are observed after injury, suggesting *de novo* expression for regenerative purposes via Wnt modulated activation of Lgr5^+^ pancreatic duct cells (Huch et al., 2015). Under Wnt activation culture conditions, murine pancreatic cells were able to generate both exocrine and endocrine structures, indicating their ability to serve as a bipotent progenitor (Huch et al., 2015). Our adult pig model has shown LGR5^+^ cells in the pancreas (Figure 1F). The pig model has historically demonstrated its relevance in pancreas research, emphasizing the importance of our LGR5^+^ pig model. To date, many differences have been reported between the mouse and human models, and many similarities have been reported between pigs and humans in this context, including the fact that pig insulin differs from human insulin by only one amino acid, and pig pancreas morphology and development is similar to humans, including in islet dispersion, which is distinct from the murine model (Lunney, et al., 2021).

Overall, significant differences have been reported between the mouse model and humans, and many similarities have been reported between pigs and humans in the gastrointestinal system. Although studies using gene-edited Lgr5 mice have made important contributions to the field and to the understanding of Lgr5^+^ cells molecular mechanisms, knowledge gaps are still highly significant from the perspective of translational medicine and alternate animal models are needed.

### LGR5^+^ cells in the cochlea

We recently emphasized the significance of the pig model in studying cochlear development, noting its close resemblance to human development, and contrasting it with the mouse model (Moatti et al., 2022). In 2014, a study revealed that, in the cochlea, new hair cells, mainly outer hair cells, are produced from supporting cells (inner pillar and third Deiters cells) expressing Lgr5 (Bramhall et al., 2014). Inhibiting Notch was found to enhance the generation of these cells suggesting that the mammalian cochlea in its early stages can regenerate hair cells after damage, with Lgr5-positive cells serving as progenitors. A subsequent study revealed that hair cell regeneration is not restricted to the cochlea but can also occur in vestibular organs (Wang et al., 2015). Neonatal mouse utricle damage triggers the activation of the Wnt target gene Lgr5 in supporting cells. These Lgr5-positive cells transform into hair cell-like cells *in vitro* and regenerate damaged hair cells *in vivo* through mitotic division and transdifferentiation pathways. Both Type I and II hair cells are regenerated, displaying stereocilia and synaptic connections. Stabilizing ß-catenin in Lgr5-positive cells enhances their mitotic activity and promotes hair cell regeneration. Lgr5 serves as a marker for Wnt-regulated progenitors of hair cells activated by damage, offering insights into mammalian hair cell regeneration mechanisms.

**FIGURE 2:**
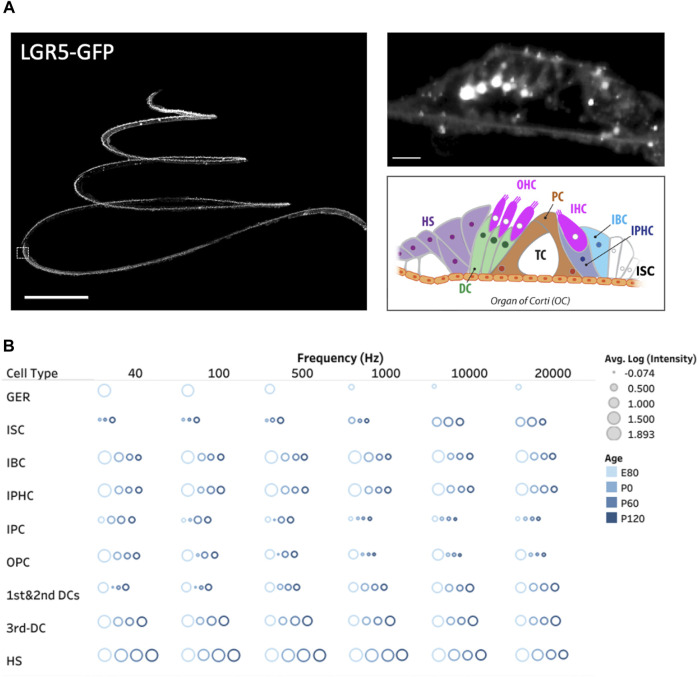
Three dimensional spatial distribution of LGR5^+^ cells in the pig cochlea and changes with age. **(A)** Maximum intensity projection (MIP) image of a transgenic porcine cochlea (P120; LGR5-H2B-GFP). Scale bar, 1,000 µm. The zoomed-in digital section where the white boxed region in the left image resides (5 µm) is shown to the right. Scale bar, 20 µm. The schematic model of cell organization in the organ of Corti is represented in the bottom. The inner sulcus cells (ISC), inner border cells (IBC), inner phalangeal cells (IPHC), inner and outer pillar cells (PCs), first, second, and third Deiters’ cells (DCs), and Hensen cells (HS). **(B)** The quantification of LGR5 expression across ages at the apex (40 and 100 Hz), middle (700 and 2000 Hz), and base (10 and 20 kHz) of the cochlea. We have reported the log of the average LGR5 intensity of each cell type across four samples/cochleae at each age and provided the individual frequencies. For this measurement in each cochlea, 10 cells from each supporting cell subset were selected and quantified per cochlea (number of cochleae, N = 4 for each age, and a total of 16 cochleae). Please note that the GER population only exists at the E80 stage.

Acknowledging the pivotal role of the LGR5 gene as an indicator for hair cell progenitors, we examined, for the first time in pigs, its expression throughout the cochlea at different frequency ranges (Moatti et al., 2022). Additionally, we explored LGR5 expression across developmental stages, including E80, P0, P60, and P120, providing valuable insights into its temporal dynamics as shown in Figure 2B. The study observed distinct patterns of LGR5 expression in various supporting cell types across different frequency regions of the cochlea in pigs. At the cochlear apex, LGR5 expression was primarily found in the greater epithelial ridge, third Deiters cells, and Hensen cells, while at the base, it was observed in all three rows of Deiters cells, Hensen cells, inner sulcus cells, and hair cells. These findings are significant as they align with increasing cochlear maturation from base to apex, suggesting a correlation between LGR5 expression and regenerative capacity. All cell types except for inner Pillar cells manifest a significant decrease in LGR5 expression with aging. Notably, Hensen cells exhibited high LGR5 expression intensity, higher than third Deiters cells, throughout the cochlea at all frequencies and different ages, a phenomenon not observed in murine models. The study also identified LGR5 expression in mature cochlear hair cells, particularly in the basal turn responsible for high-frequency sound detection. The reported differences between species, coupled with the current lack of detailed human data, underscore the value of the pig model in cochlear research.

### LGR5^+^ cells in the skin

LGR5 has been identified in the bulge region of hair follicles in mice, humans, and pigs (Polkoff et al., 2022b). While the murine model has offered valuable insights into the localization and molecular basis of LGR5^+^ cells within the follicle, significant anatomical and physiological differences between mice and humans—such as contrasting coat characteristics, growth periods, and skin attachment—highlight the necessity of selecting a suitable model for preclinical studies. Further, studies on wound healing have shown a 78% similarity between pigs and humans compared to 53% between humans and mice (Sullivan et al., 2004), reinforcing the pig skin’s resemblance to human skin, particularly in aspects like the epidermis, hair follicle count, thickness, and microflora (Lunney et al., 2021). This similarity extends to pharmacology and toxicology research as well (Lunney et al., 2021). Given that burn-related injuries can lead to mortalities through infection, fluid loss, and pH imbalances, leading to organ failure, the relevance of the transgenic LGR5^+^ pig model becomes even more significant, particularly considering the roles of LGR5^+^ cells in microbial responses and regeneration (Sigal et al., 2017; Huch et al., 2015; Lin et al., 2017).

**FIGURE 3:**
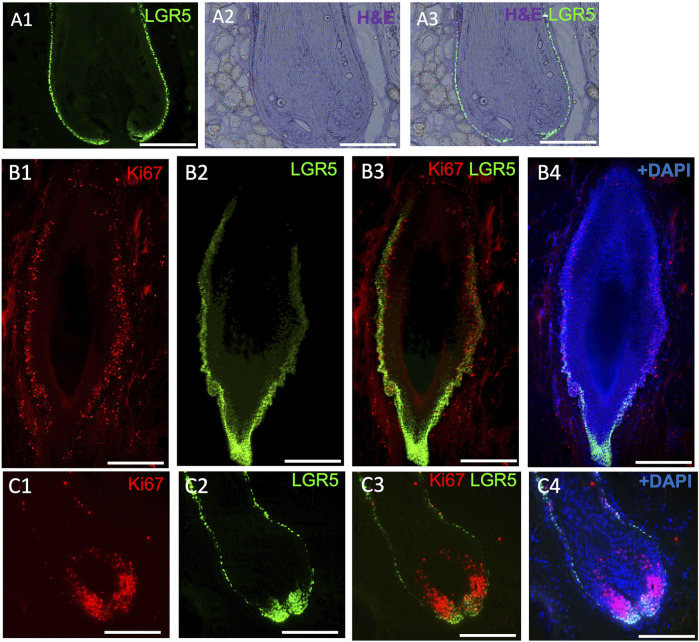
LGR5 expression by hair follicle stem cells (HFS) and how they are impacted in chronic wounds. (A1–A3) LGR5 expression can be observed in the hair follicle at the base and bulge region on a healthy hair follicle, where HFS resides. Note single layers of LGR5^+^ cells in a healthy hair follicle. Scale bar, 125 uM. (B1–B4) LGR5^+^ cells in hair follicles surrounding chronic wounds. Note abnormal morphology and an increase of proliferation in the base and bulge region, indicated by KI67 staining. Scale bar, 125 um. (C1–C4) In contrast, follicles from healthy skin have reduced proliferating LGR5^+^ HFS in the bulge region. Scale bar 200 um.

In our studies, we have detected LGR5^+^ cells in the skin using the transgenic pig model (Figure 3A), prompting us to investigate their location in naturally occurring chronic wounds in adult pigs. We have observed an abnormal phenotype (Figure 3B) and increased proliferation in the bulge region of hair follicles surrounding chronic wounds (Figure 3C). Previous research in mice has shown that after a skin wound, Lgr5^+^ cells and their progeny migrate to the site and participate in the wound healing process (Ito et al., 2005; Joost et al., 2018). Although direct studies of LGR5^+^ stem cells in human wound healing have not been possible, clinical observations indicate that areas with hair heal faster than hairless regions (Jimenez et al., 2015). A small, randomized control trial supported this by showing better healing in chronic leg ulcers with grafts from follicle-containing areas compared to non-hairy regions (Martínez et al., 2016).

Due to the similarities between human and pig skin, and the inability to reliably detect human LGR5 cells by immunohistochemistry makes the LGR5-H2B-GFP pig uniquely suited to understand the responses of LGR5 hair follicle stem cells after injury. However, at this time, our current model lacks lineage tracing capabilities, leaving gaps in understanding the role of these cells post-differentiation. To address this, the development of a lineage tracing model is crucial, as it will help elucidate the mechanisms underlying the effects of LGR5^+^ cells after injury.

### LGR5^+^ cells in the lungs

Adult lung tissue is composed of various epithelial cell types, and distinct cell populations are involved in both homeostasis and injury repair of the adult lung epithelium, depending on their anatomical location (Barkauskas et al., 2013; Lee et al., 2017; Zepp et al., 2017; Leeman et al., 2019). In addition, different stromal factors regulate the behavior of these cells (Lee et al., 2017). However, there are many anatomical differences between mouse and human lungs including the lack of basal cells in intrapulmonary airways, the presence of respiratory bronchioles in humans but not mice, and a lower abundance of bronchioalveolar stem cells in mice than humans, among others (Weibel and Gomez, 1962; Liu et al., 2019; Salwig et al., 2019). As a result, the mouse, while invaluable for basic research, has significant limitations for translational research. In contrast, the pig respiratory system has many important similarities to that of humans, including the presence of basal cells in the intrapulmonary airways, and respiratory bronchioles, making pigs a relevant model for the investigation of respiratory diseases, drug development and immune responses (Lunney, et al., 2021).

**FIGURE 4:**
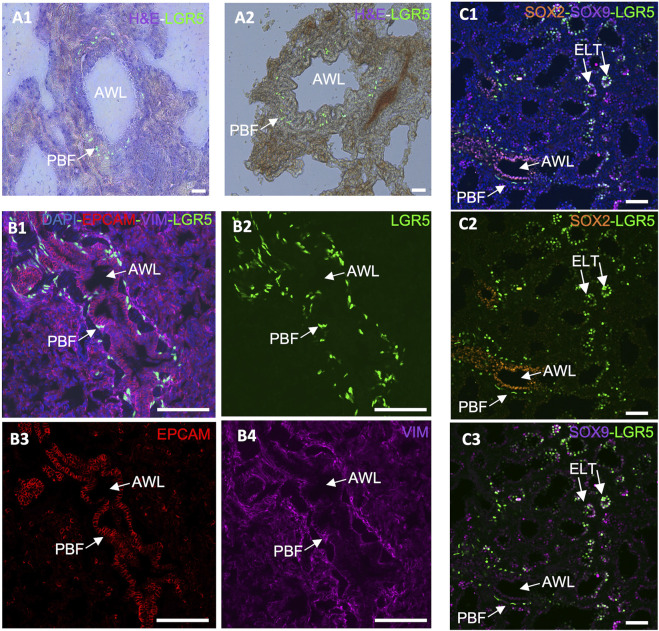
Expression of LGR5 cells in the adult and developing pig lung. (A1–A2) GFP and H&E counterstain, showing the location of LGR5 cells surrounding the airway lumen (AWL) in the peribronchial region in an adult pig. Scale bar, 50 um. (B1–B4) Cross section of an adult airway and surrounding alveoli showing the peribronchial location of the LGR5^+^ cells and the mesenchymal phenotype identified by lack of EPCAM and presence of VIM. No LGR5^+^ EPCAM^+^ cells are present in adult pigs. (C1–C3) Lung of pig fetus at gestational day 80 (E80) showing the presence of both mesenchymal LGR5^+^ cells adjacent SOX2^+^ airway epithelium, and LGR5^+^ SOX9^+^ cells in the epithelial cells of the tip of the elongating lung airways. Scale bar, 50 um.

In the adult lung of humans, mice and pigs, a distinct subset of LGR5^+^ mesenchymal cells have been found (Lee et al., 2017; Polkoff et al., 2022a). In mice, these cells are located in the alveolar compartments, where they stimulate alveolar differentiation of epithelial progenitors via Wnt activation (Lee et al., 2017). Conversely, in pigs and humans, these cells reside adjacent to the airway epithelium, in a sub-basal or peribronchial region (Figure 4A) (Polkoff et al., 2022a). This contrast is pivotal as it suggests that the role and behavior of these cells are context-specific and cannot be simply extrapolated from one species to another. The advent of single cell RNAseq and its application to the study of the healthy and diseased lung in humans has allowed for a greater understanding of the complex signaling pathways between mesenchymal and epithelial cells that control development events, as well as repair after injury and cancer cell transformation (He et al., 2013; Koenitzer et al., 2020; Salcher et al., 2022; Blumhagen et al., 2023; Sikkema et al., 2023). Results to date show support for the differences between mice and humans and the pig/human similarities.

Taking that into consideration, the anatomical difference in the location of LGR5^+^ mesenchymal cells among mice, pigs and humans suggests that these cells may play a distinct role in these species. For instance, in pigs, the close association of LRG5^+^ peribronchial fibroblasts with sensory nerve fibers (Polkoff et al., 2022a), raises questions regarding their effects on nerve fibers and surrounding cells. Furthermore, co-culture organoid systems have unveiled disparities in the behavior of LGR5^+^ mesenchymal cells in mice and pigs. While the mouse Lgr5^+^ cells increase alveolar organoids formation (Lee et al., 2017), pig LRG5^+^ peribronchial fibroblasts (PBF) have shown the ability to support formation of bronchiolar organoids (Polkoff et al., 2022a), supporting the assertion that LGR5^+^ cells exhibit different functions in different species. As cells’ abilities to form organoids have been associated with their ability to display important epithelial repair (Kong et al., 2021), it may be hypothesized that LRG5^+^ peribronchial fibroblasts play a distinct role in lung epithelial repair Considering the observed phenomenon in the murine model and the established association between the Wnt signaling pathway and Lgr5^+^ cells in the pulmonary systems of various species, it is reasonable to postulate the potential applicability of a similar mechanism following lung injury.

Furthermore, while in the postnatal lung epithelial LGR5 expression is absent, it is present during fetal development in the tip of the elongating lung buds (Figure 4C), and can be reactivated in bronchoalveolar organoids originating from adult basal airway cells (Polkoff et al., 2022a). This supports a responsiveness of LGR5^+^ cells to their niche and to environmental cues and contributes to the hypothesis that they may be involved in a lung injury response. According to unpublished results from our research group, R-spondin 2 (RSPO2) and R-spondin 3 (RSPO3), which participate in the Wnt signaling pathway and activate LGR5^+^ cells as either expressed by the mesenchymal LGR5 cells or by surrounding stromal cells (data not shown). As it has been previously reported that RSPO2 plays an important role in lung epithelial stem/progenitor cell homeostasis and regeneration, and it may therefore be a potential therapeutic target for chronic lung disease treatment (Raslan et al., 2022).

Overall, numerous disparities have emerged in lung Lgr5 cell expression patterns among the mouse model, when compared to humans and pigs. These findings underscore the significance of the pig model in translational research. There are multiple molecular mechanisms that remain yet to be explored in the pig model and that could potentially pave a way for new discoveries in the field.

### LGR5^+^ cells in the musculoskeletal (MSK) system

**FIGURE 5:**
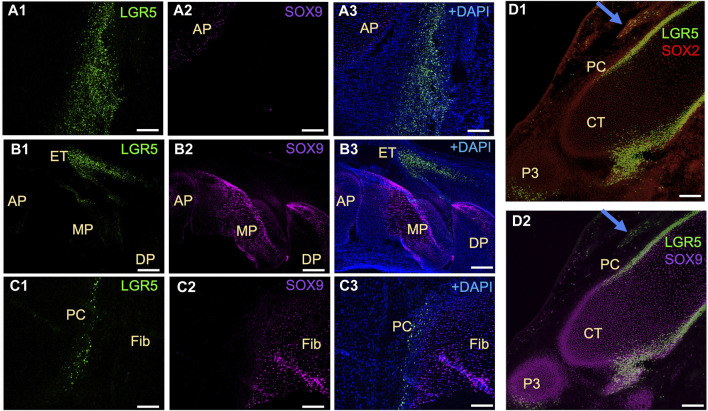
Distribution of LGR5^+^ cells in developing musculoskeletal tissues of the fetal LGR5-H2B-GFP pig. (A1–A3) In the knee joint at E50 of fetal development, the anterior patella (AP) shows LGR5^+^ and few SOX9^+^ cells in the cartilage region. (B1–B3) In the hoof, LGR5^+^ cells can be seen in the extensor tendon (ET) over the middle phalanx (MP). LGR5 cells do not express SOX9 and SOX9^+^ cells are more abundant in the middle phalanx and distal phalanx (DP). (C1–C3) In the head of the fibula (Fib), SOX9^+^ cells are present in the cartilage region, and LGR5+ cells can also be observed in the perichondrium (PC) overlying this area. (D1–D2) At E80, LGR5+ cells can be observed in the developing hoof adjacent to the middle phalange and in the distal portion of the distal phalange. Additionally, LGR5^+^/SOX2^+^ cells can be seen in the developing tendon (blue arrow). Scale bar, 50 um.

In mice, Lgr5^+^ cells play a crucial role in the formation of joint tissues such as the cruciate ligament, synovium, and articular cartilage. These cells are also capable of promoting joint healing post-injury (Feng et al., 2019). Furthermore, LGR5 expression is found in the deep zone of articular cartilage in humans diagnosed with osteoarthritis (Lee et al., 2021). Utilizing the LGR5^+^ H2B-GFP pig model, our research focused on the presence of LGR5^+^ cells during joint embryonic development. Notably, LGR5^+^ cells were observed in the tissues surrounding the anterior and distal patella, hoof, and head of fibula (Figure 5). Additionally, SOX9 staining revealed the presence of this key cartilage transcription factor in these tissues (Figure 5 A2, B2, C2, D2). Postnatally, in the juvenile pig (1 month), LGR5^+^ cells were located within the connective tissue/synovium directly adjacent to the menisci (Figure 5B), in the tendon/ligament sheath and in smaller numbers in between the fascicles of the tendon (data not shown). In contrast, in the juvenile mouse, 10 days after birth, Lgr5 is undetectable in either the articular cartilage or the meniscus (Feng et at., 2019). However, as the healthy pig ages the numbers of LGR5^+^ in the joints, bone and ligaments also drastically decreases and in a 25-month-old LGR5^+^ H2B-GFP boar, LGR5^+^ cells are essentially undetectable in the MSK system (data not shown). In the muscle, Lgr5 cells function as an adult progenitor cell capable of regeneration (Leung et al., 2020). Moreover, cells were co-expressing LGR5^+^ and SOX9, which is a transcription factor that plays a crucial role in the development of various tissues, including cartilage and bone. SOX9 regulates the expression of several genes involved in chondrogenesis, including collagen type II and aggrecan, which are major components of cartilage matrix (Lefebvre et al., 2019). Furthermore, Sox9 has been shown to be co-expressed with LGR5 in the lung and gastrointestinal tissues (Polkoff et al., 2022a; Schaaf et al., 2023).

Additionally, mesenchymal stem cells isolated from a LGR5^+^ H2B-GFP adult pig were differentiated into chondrogenic lineage, and the differentiating cells upregulated LGR5 expression as detected by the increased GFP signal (data not shown). The fact that the cells reactivated LGR5 to undergo chondrogenic differentiation open a field for the investigation of LGR5 role in chondrogenic tissue formation. Given how little is known about the role of LGR5^+^ cells in joint development and after injury, that the pig is a rising animal model for regenerative medicine and tissue engineering in the musculoskeletal system (Cone et al., 2017), the LGR-H2B-GFP pig presents a unique opportunity to begin to address these critical translational issues.

### LGR5^+^ cells in the eye

Pigs are a suitable animal model for ophthalmological studies due to their important similarities to humans in this area, as reviewed by Lunney et al. (2021). Among these similarities, the presence of holangiotic retinal vasculature, cone photoreceptors in the outer retina, scleral thickness, a lack of tapetum, corneal collagen fibrillar arrangement and evenly distribution of goblet cells throughout the conjunctiva, are of particular importance (Lunney, et al., 2021).

**FIGURE 6:**
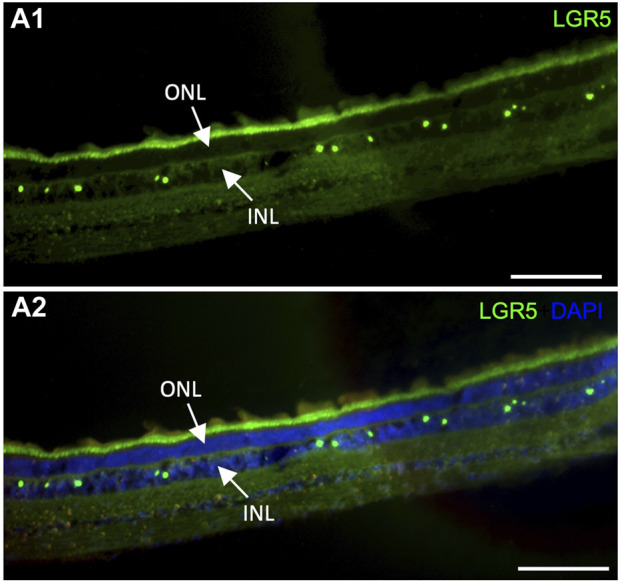
Expression of LGR5^+^ cells in the adult pig retina. (A1–A2) As has been reported in mice (Sukhdeo et a., 2014) Lgr5^+^ cells are expressed in the inner nuclear layer (INL) of the retina but not on the outer nuclear layer. In mice, the Lgr5^+^ cells have been identified as glycinergic amacrine cells. Scale bar, 100um.

It has been previously suggested that Lgr5^+^ amacrine cells may act as a regenerative source due to their role in generating new retinal cells, supporting an interesting field of studies for new regenerative therapeutic approaches for retinal degenerative diseases (Chen et al., 2015). In humans, LGR5 expression was previously demonstrated in healthy and inflamed human lens epithelial cells. Interestingly, expression was reduced after inflammation, which was correlated with the fact that LGR5 may be considered a stemness marker that is affected by inflammatory damage at an early stage (Curcio et al., 2015). By the evaluation of our pig model, it was possible to observe LGR5^+^ cells in the retina arranged in a “zigzag” manner (Figure 6A), similarly to previous observations in adult mice (Chen et al., 2015). Hence, LGR5^+^ cells hold promise as an important tool in regenerative medicine, and the pig represents a suitable model for advancing translational research in ophthalmology.

## Conclusion

LGR5 exhibits important functions throughout the body in numerous tissues in diverse contexts, such as in during development, organ homeostasis and repair after injury. LGR5^+^ cells depend on ligands from their niche to determine cell fate, contributing to either regular tissue turnover, regeneration after injury or even cancer progression. Even though many studies have been performed throughout the past years, numerous issues remain unclear regarding relevant molecular mechanisms, niche complexity, cell crosstalk and potential target molecules for disease treatment. In this review we have summarized the similarities and differences between mice, pigs, and humans with respect to LGR5. This includes differences in distribution and temporal expression of LGR5 in the lung (Polkoff et al., 2022a), skin (Polkoff et al., 2022b), cochlea (Moatti et al., 2022), and the intestines (Schaaf et al., 2023). Hence, the roles and behaviors of these cells are context-specific and cannot simply be extrapolated from one species to another.

The development of a transgenic large animal model creates the possibility to perform a cross-species evaluation of all relevant body tissues, which highlights an exciting field of studies providing new avenues for future research, especially with regard to translational medicine. A current limitation of our model is its lack of lineage tracing capability. While previous mouse studies have provided valuable lineage tracing analyses revealing the roles of Lgr5^+^ cells in tissue development across various tissues, our model currently only allows the study of LGR5 cells, not their differentiated progeny. Notwithstanding this weakness, the LGR5-H2B-GFP pig allows the study of tissue distribution and transcriptome changes of LGR5 cells during development, homeostasis and after injury, allowing cross-species evaluation in relevant body tissues. A deeper understanding of niche requirements, cell metabolism, differentiation and proliferation pathways, and molecules influencing cell fate is essential to developing new and effective regenerative therapies. The ongoing advancements in this field promise to enhance further investigations and progress, laying the groundwork for future research on the regenerative properties of LGR5 stem cells, cancer treatment, and drug development.
